# Surface-dependent quenching of Qdot emission can be a new tool for high resolution measurements

**DOI:** 10.1038/s41598-023-28910-8

**Published:** 2023-02-01

**Authors:** Kaoru Okura, Hitoshi Tatsumi

**Affiliations:** Department of Applied Bioscience, Kanazawa Institute of Technology (KIT), Yatsukaho 3-1, Hakusan-shi, Ishikawa 924-0838 Japan

**Keywords:** Biological techniques, Imaging, Microscopy, Sensors and probes, Optical spectroscopy

## Abstract

Single quantum dots (Qdots) are often used in the field of single-molecule imaging. Qdots are sensitive to changes in the physical interactions between the Qdots and the surrounding materials. However, the spectral changes in a single Qdot emission have not been studied in detail. Low-temperature plasma treatment of glass surfaces reduced the intensity of the 655 nm emission peak of Qdot655 on glass surfaces, but did not significantly change the intensity of the 580 nm emission. Silanization of the glass surface increases the thickness of the silane layer, and the 655 nm emission peak increased. When single Qdots on the untreated glass were imaged, plasma treatment decreased the intensity of red emission and increased yellow emission. When Qdots were brought close to the glass surface in the range of 28–0 nm, the red emission intensity decreased and the yellow emission intensity increased slightly. When single actin filaments were labeled with Qdots, fluctuations of the yellow and red emission of the Qdot were detected, which reflected the very small distance changes. Our results indicate that the local interaction of Qdots with the glass surface improves the spatial and temporal resolution of optical measurements of biomolecules labeled with Qdots.

## Introduction

Semiconducting quantum dots (Qdots) have been shown to possess several photophysical properties that are superior to those of organic fluorophores: high-absorption cross sections, excellent photostability, broad excitation spectra, and narrow emission spectra^1^. Multiple peaks in the emission spectra of Qdots with a single light source have been reported^2,3^. These peaks are affected in a different way, e.g., by the coupling of core–shell^4^, chemical compounds^5^, ions^6,7^, air–water surface interface^8^, and surface plasmon resonance^9^. The quenching of Qdots (CdSe) by gold nanoparticles in the vicinity was examined in detail; the distance required for the quenching is estimated at 7.5 nm. These reports support the idea that Qdots are sensitive to changes in the surrounding medium, chemical compounds, or physical interactions between the surface of Qdots and the environment (or surrounding materials). The spectral change in single Qdots can be occurred by changes in the size of the Qdots and formation of emissive-defect site. In the case of the defect formation, a broad emission spectrum from the defect-site should be observed at longer wavelength compared to the band-edge emission (usually the main emission peak)^10^. The Qdots photoluminescence quenching is ascribed to several mechanisms, including non-radioactive recombination pathways, inner filter effects, electron transfer processes, and binding interactions^3,11–13^. A preceding study^14^ reports quenching of Qdots (CdSe) by gold nanoparticles at the single molecule level; the peak of the photoluminescent spectrum (520 nm) declined without an apparent longer wavelength broad emission, and distance (ca. 7.5 nm) dependent quenching was observed, suggesting the quenching is due to FRET between Qdots and nanogold particles.

Spectrally-resolved fluorescence imaging of single CdSe/ZnS Qdots that is charged by electrospray deposition under a negative bias has revealed a net blue shift in Qdot emission^15^, and a recent study^16^ has shown that the spectrum consists of multiple components and a certain component is selectively affected by electron transfer from the Qdot to the surface materials. These studies suggest the spectra of Qdots may be changed by the glass-surface treatment and by the distance between the Qdot and the glass surface. The plasma treatment of the glass surface removes the adsorbed organics and dust particles of a few nm thickness and the glass surface atom is terminated with hydroxyls^17^, which may quench the Qdots near the surface or change the emission spectrum.

The spatial resolution of conventional light microscopy with a high numerical aperture lens is about 300 nm. The penetration depth of the total internal reflection microscopy (TIRM) along the z-axis is about 100 nm ^18^. High-resolution (20 nm) fluorescence photoactivation localization microscopy (FPALM) analyzes thousands of images of single fluorophores per acquisition, which requires time-consuming procedures^19^. Thus, distance-dependent spectral changes in Qdots may provide an opportunity to significantly improve the spatial and temporal resolution of optical measurements. Single Qdots are often used in the field of molecule imaging^20^. However, the spectral change of single Qdots on the glass surface has not been examined in detail, although they have been used for single molecule imaging in the field of biophysics.

In this study, we examined the effect of glass-surface treatment on the spectra of streptavidin-conjugated Qdots. The Qdot − surface distance-dependent changes in the quantum dot emission in a single Qdot level were analyzed. Single actin filaments were labeled by the Qdots and the spectral changes of the Qdot emission were analyzed for the first time, which suggest the local interaction between the Qdot and the glass surface improves the spatial and temporal resolution of the optical measurement of biomolecules labeled with Qdots.

## Results

### Plasma treatment and silanization of the glass surface change the emission spectra of Qdots in an opposing way

**Figure 1:**
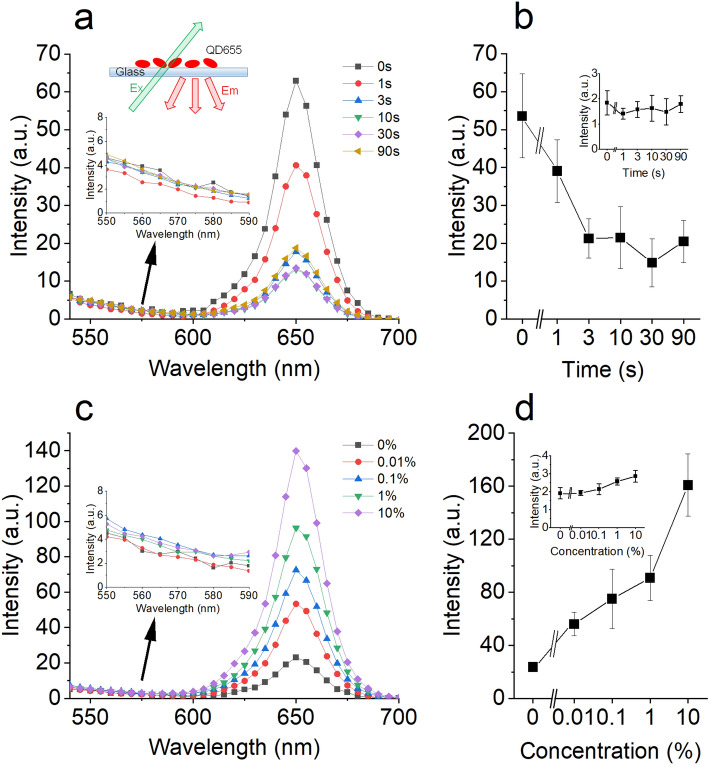
The effect of glass surface plasma treatment on the emission spectrum of QD655. (**a**) Emission spectrum of QD655 on the glass surface at different durations of plasma treatment. Excitation at 530 nm. Upper inset schematic illustration of the setup. Lower inset, the magnification of the yellow part of the spectrum. (**b**) The peak emission intensity of QD655 decreases with increasing the duration of plasma treatment (n = 3). Inset, the emission intensity at 580 nm does not change with increasing the duration. (**c**) Emission spectrum of Qdot655 on the glass surface treated with different concentrations of the silane coupling agent. Excitation at 530 nm. Inset shows the magnification of the yellow part of the spectrum. (**d**) The peak emission intensity of Qdot655 increases with increasing the concentration of the silane coupling agent (n = 3), but the emission intensity at 580 nm does not change (inset). The vertical bars in (**b**) and (**d**) denote the standard deviation.

Changes in the emission spectra of Qdots plated on the plasma-exposed glass surface were examined, as shown in Fig. 1. Qdot655 had an emission peaked at 655 nm when excited by 530 nm light. The intensity at 655 nm decreased to 40% of the control (no plasma treatment) with increasing plasma treatment time, while the intensity between 545 and 600 nm did not change significantly (Fig. 1). No obvious long-wavelength emission (longer than 655 nm) was observed, and the peak emission wavelength (655 nm) was not changed apparently.

Attenuation of the maximum of the fluorescence emission spectra at different durations of plasma treatment showed the irradiation-duration-dependent attenuation reached maximum at 30 s and half-maximum attenuation at 1−2 s, while the intensity of emission at 580 nm was not changed under these conditions (Fig. 1b). Nearly the same changes in the emission spectra of Qdots were observed for Qdot565, Qdot585, Qdot605, and Qdot705 (Figure S1).

**Figure 2:**
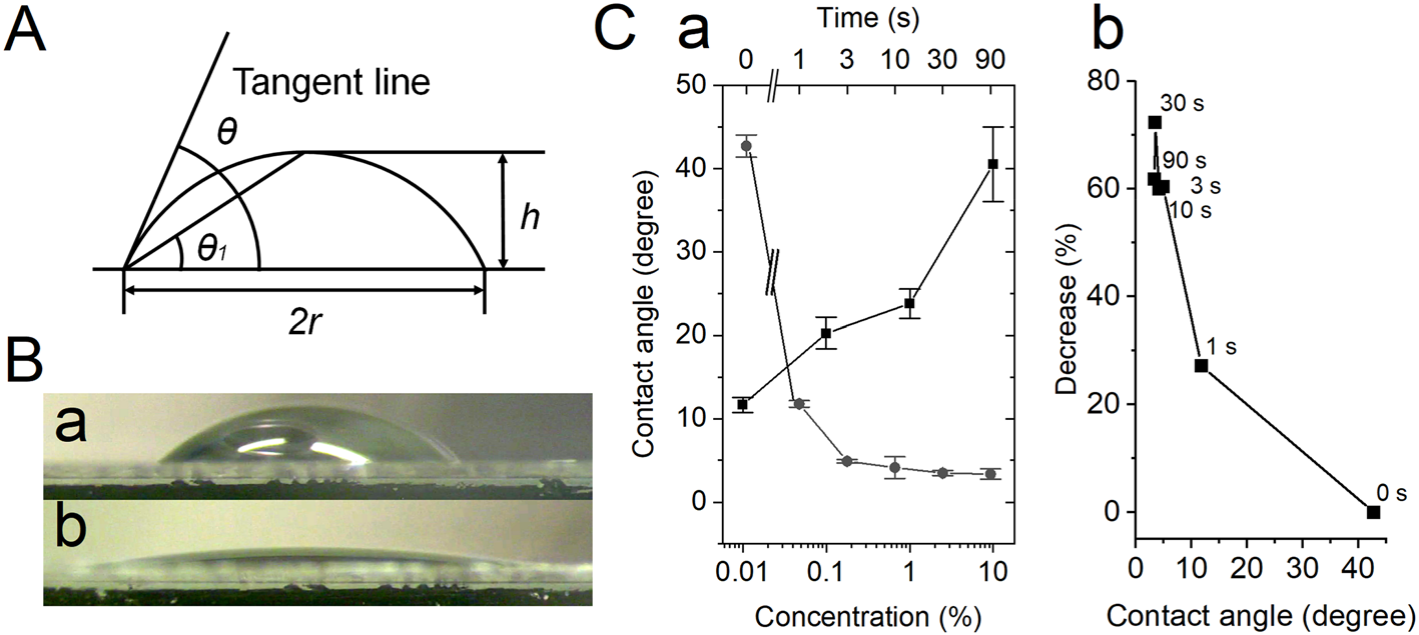
(**A**) Schematic diagram of the contact angle (θ) and the measurement of θ1. (**B**) A side view of the water droplet (30 μl) on untreated glass (**a**) and on glass with low-temperature plasma treatment (30 s) (**b**). (**C**) (**a**) The relationship between the contact angle (θ) and the duration of low-temperature plasma treatment (circle) and the relationship between θ and the concentration of the silane coupling agent (square) (n = 3). (**b**) The relationship between the contact angle (θ) and the percent decrease in the peak intensity of the emission spectrum of Qdot655. Numbers denote the duration of plasma treatment. The duration of plasma treatment is shown above the symbols.

Low-temperature plasma treatment can increase the wettability of the glass surface^21,22^. The water contact angle (WCA) of the substrates before the plasma treatment was 44°, and plasma treatment significantly reduced the WCA to values less than 10°. The effect of low-pressure plasma treatment on the WCA depended on the duration of the plasma treatment, as shown in Fig. 2C. The Qdot emission decreased with increasing the wettability of the glass surface, as shown in Fig. 2Cb.

TEM imaging shows silanization increases the silane layer thickness (ca. 28 nm)^23^, and possibly increases the distance between Qdots and the plasma-treated glass surface. The emission peak at 655 nm on the plasma-treated glass surface increased by increasing the concentration of the silanization agent in a dose dependent manner (Fig. 1C), and silanization increased the contact angle on the glass surface (Fig. 2C). These results suggest that surface hydrophilicity affects the peak emission of Qdots, but does not affect the emission at shorter wavelengths.

### Analysis of single Qdots emission on the modified glass surface

The spectrum of Qdots mentioned in the above section is an ensemble of the spectra of individual Qdots. In other words, the overall spectrum is supposed to be a superposition of individual Qdots. Emission from a single Qdot is often used to analyze the behavior of biological molecules ^20^. The emission change of single Qdots is examined in the following sections in order to study the mechanism behind the spectrum change and to find its usage in the imaging of biomolecules.

**Figure 3:**
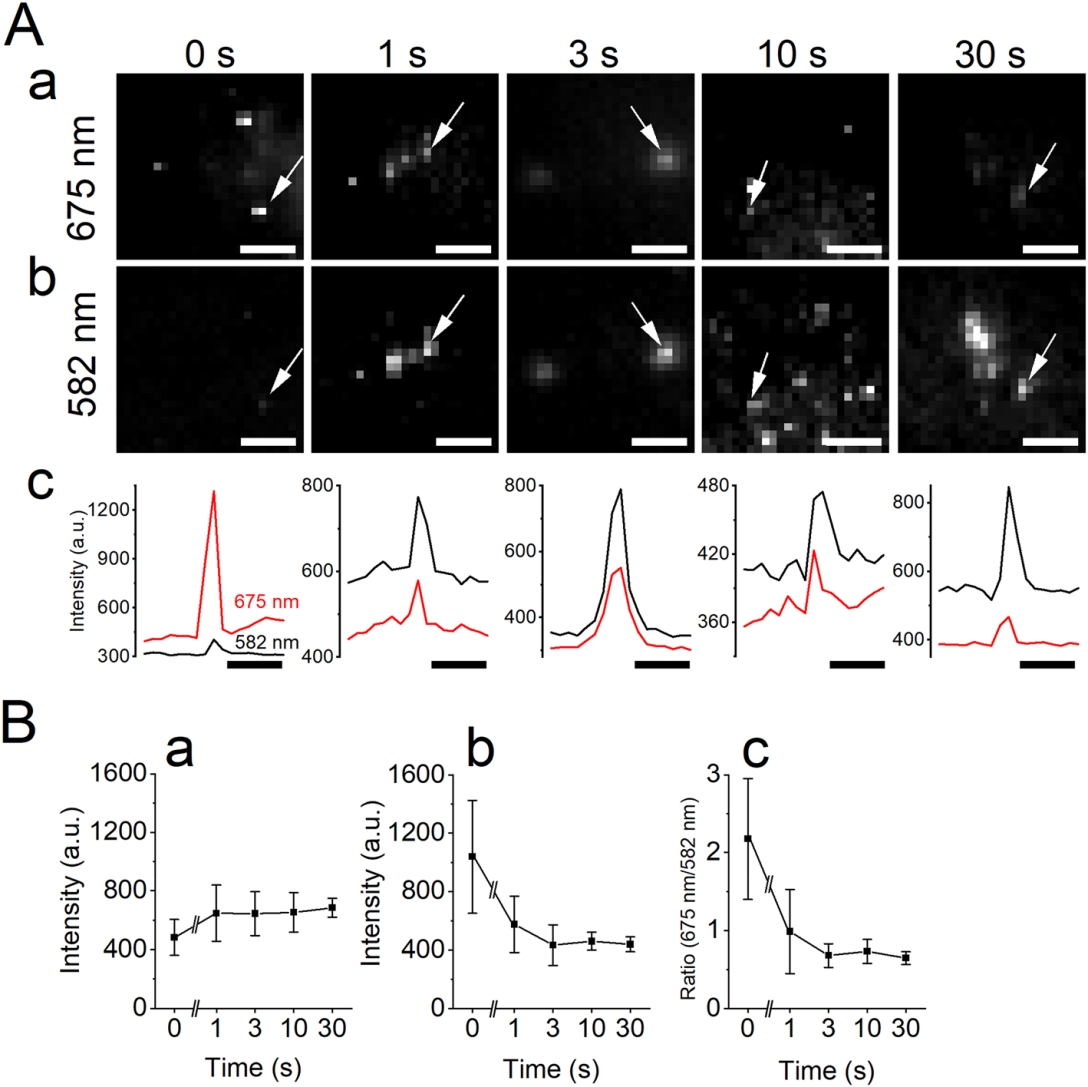
Single-Qdot analysis of the effect of the low-temperature plasma treatment. (**A**) Images of Qdot655 on different durations of the plasma treatment (0–30 s) through the red filter (a, center wavelength 675 nm) and yellow filter (b, center wavelength 582 nm). The arrows point to the single Qdot measured in (**c**). (**c**) Intensity profile of the Qdot in panels (**a**) and (**b**). (B) Emission intensity changes dependent on the duration of the plasma treatment; (**a**) yellow emission intensity, (**b**) red emission intensity, and (**c**) ratio of the yellow and red emission intensities. Bars, 5 μm.

The changes in the yellow (photoluminescence passed through a band-pass optical filter 550–614 nm, centered at 582 nm, short-wavelength foot of the emission profile) and red (642–708 nm, centered at 675 nm) intensities of the emission spectra of a single quantum dot on the plasma-exposed glass surface were examined, as shown in Fig. 3. As shown in Fig. 1, the yellow and red emission intensities correspond to the part of the spectrum that has low and high sensitivity to the modification of the glass surface (plasma treatment and silanization). When single Qdots on the untreated glass (without plasma treatment) were imaged using a TIRF-microscope, the intensity of the red emission was high compared with that of the yellow emission. When the glass surface was treated with the low-pressure plasma, the intensity of the red emission intensity decreased, while the yellow emission intensity was not changed markedly. The decrease in the red intensity was dependent on the duration of plasma treatment (Fig. 3). The ratio of the red/yellow emission intensity decreased from 2.2 to 0.6, which showed a similar tendency of change to the emission spectra of Qdot655 (Fig. 1).

**Figure 4:**
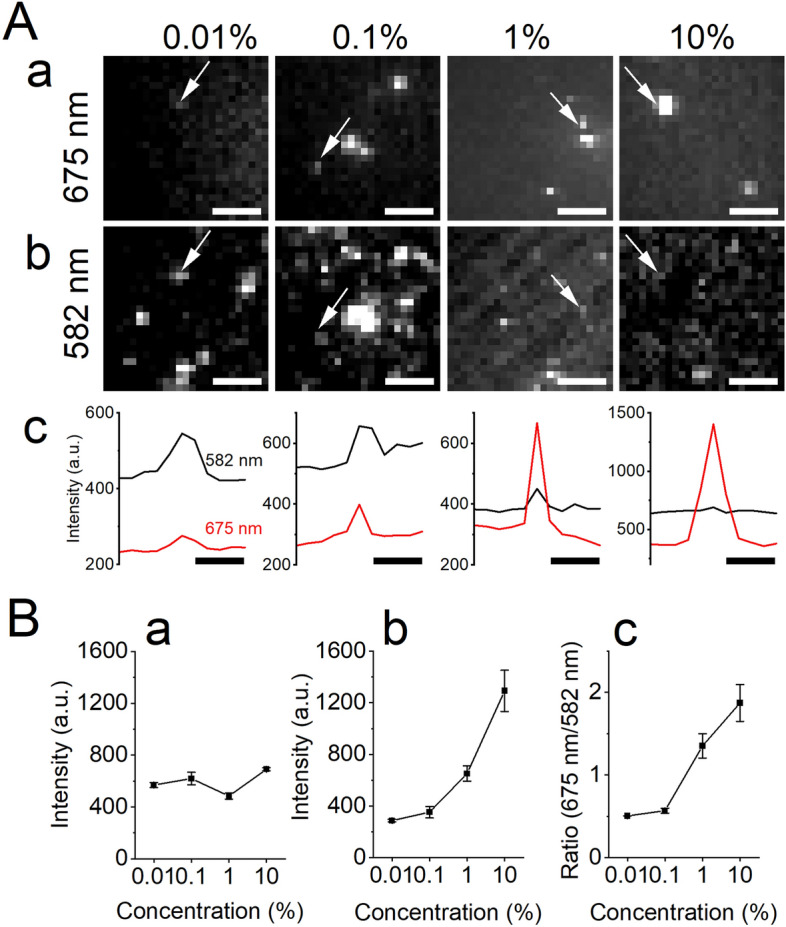
Single-Qdot analysis of the effect of treatment with different concentrations of the silane coupling agent. (**A**) Images of Qdot655 on different concentrations of the silane coupling agent through the red filter (a, center wavelength 675 nm) and yellow filter (b, center wavelength 582 nm). The arrows point to the Qdot measured in (**c**). (**c**) Intensity profile of the Qdot in (**a**) and (**b**). (**B**) Emission intensity changes dependent on the percent of the agent; (**a**) yellow emission intensity, (**b**) red emission intensity, and (**c**) ratio of the yellow and red emission intensities. The notation of this figure is the same as Fig. 3.

Silanization of the glass surface increased the red emission intensity of a single Qdot655 emission on the plasma-treated glass surface, while the yellow emission intensity did not change markedly (Fig. 4). This effect of silanization was dependent on the concentration of the silanization agent (Fig. 4B). Thus, the red and yellow emission intensities of an individual Qdot changed according to the plasma treatment and silanization in an opposite way to that seen in the spectral changes in Fig. 1.

The intensities of the individual Qdots were different, as shown in Figs. 3 and 4, presumably due to that (1) the distance between the individual Qdot and the glass surface was different, (2) the number of Qdots in the individual spot was different, and (3) the longitudinal axis of individual Qdots was not the same as the polarization axis of the laser illumination. The yellow and red intensity of the spots, however, changed in the same way, as shown in Fig. 3 and 4. Single Qdots showed blinking. The blinking of the red and yellow emission intensities was observed in the β -mercaptoethanol free solution, and they blinked in synchrony. Simultaneous quenching of both was also detected. These observations are not apparent from ensemble measurements of many Qdots using a fluorospectrometer.

### Qdot − glass surface-distance-dependent changes in the emission of the Qdot

**Figure 5:**
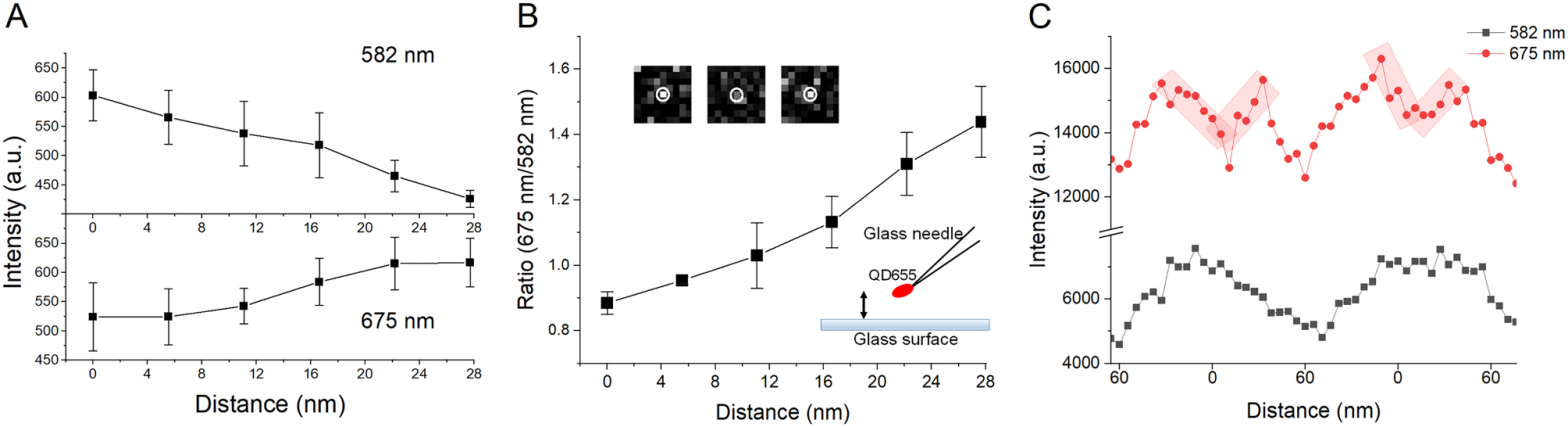
Qdot and glass surface distance-dependent changes in the emission intensity. (**A**) upper panel shows the distance-dependent decrease in the yellow emission intensity of the emission intensity (center wavelength 582 nm) and in the red emission intensity (center wavelength 675 nm). One-way ANOVA analysis shows significant difference among the emission intensities (*p* < 0.01 for 582 nm) and (*p* < 0.05 for 675 nm), which decreased and increased respectively with increasing the distance between the QD and the glass surface (Fig. 5A). The distance dependence of the 582 nm emission intensity shown in the upper chart of Fig. 5A could be explained by the spatial change of the evanescent light intensity (space constant of evanescent wave, 100 nm). The ratio between the 582 and 675 nm emissions is mainly dependent on the very short distance (27 nm) dependent quenching of Qdots. (**B**) The ratio of yellow/red emission intensity increases with increasing the distance. Insets are images of the emission of a Qdot at 28 nm above (left), on the surface (center), and 28 nm above (right), and schematic illustration of the experimental setup. (**C**) Distance-dependent changes in the red (red circles) and yellow (black circles) emission intensities. Qdots were moved 60 nm up and down repetitively every one sec. The tip approached from 60 nm above the surface to the surface (0 nm) two times. The horizontal axis shows the position of the Qdot from the surface of glass and the vertical axis shows the emission intensity of Qdot655. The Qdot attached on the tip of glass capillary would not change direction, so this Qdot is used to estimate the distance-dependent changes in the emission. The tip of the glass capillary affects the evanescent field. The fine glass capillary (approximately 1 μm) was used in panel A and slightly larger glass capillary (approximately 2 μm) was used in panel C. This difference and the direction of longitudinal axis of the Qdot at the tip of the capillary would affect the signal level of these data (see more in discussion) and the amplitude of the oscillation in panels A and C.

The distance-dependent changes in the emission intensity of Qdot655 were examined by displacing Qdots from a plasma-treated (30 s) glass surface. The position of a small number of Qdots at the tip of the glass capillary was changed using a piezo actuator (Fig. 5B inset), the Qdots were illuminated by the total internal reflection of 532 nm laser light, and the position-dependent change in the intensity of emission was detected through the yellow and red band pass filters. The amplitude of the displacement of Qdots at the tip of the glass fine capillary was 27.7 nm. When the tip gradually approached the glass surface, the red emission intensity declined, while the amplitude of the yellow one increased (Fig. 5A). A similar experiment was repeated using the larger amplitude of displacement (60 nm). When the tip approached the glass surface, the yellow emission intensity increased. The intensity of the red one also increased when the tip approached the glass surface from 60 to ca. 20 nm, but it decreased by 30–40% from the peak value when it more closely approached the surface (20–0 nm) (Fig. 5C, shadowed in red). The intensity of the emission changed in the opposite way when the Qdot moved away from the surface. These changes were repeatedly observed (the tip was repeatedly moved 60 nm up and down every sec), suggesting the spectral changes of the Qdots emission was reversible. In summary, the intensities of the yellow and red emissions increase with a space constant for the exponential increase of ca. 100 nm when the Qdot enters the evanescent field of illumination in the range 20–100 nm from the surface of the glass^24^. The intensity of the red emission decreased when the Qdots entered in the range 0–20 nm from the surface, but that of the yellow emission did not.

### Imaging of Qdots bound to actin filaments

Actin is the most abundant protein in most eukaryotic cells. Our recent study suggested that cofilin (actin filament-severing protein) protein, prefers to bind actin filaments with less tension and that actin filaments with less tension showed larger fluctuations^25^, and positive correlation between the index of fluctuation and the on-rate of cofilin binding is reported^18^. The fluctuations of actin filaments near the glass surface was examined with Qdots.

**Figure 6:**
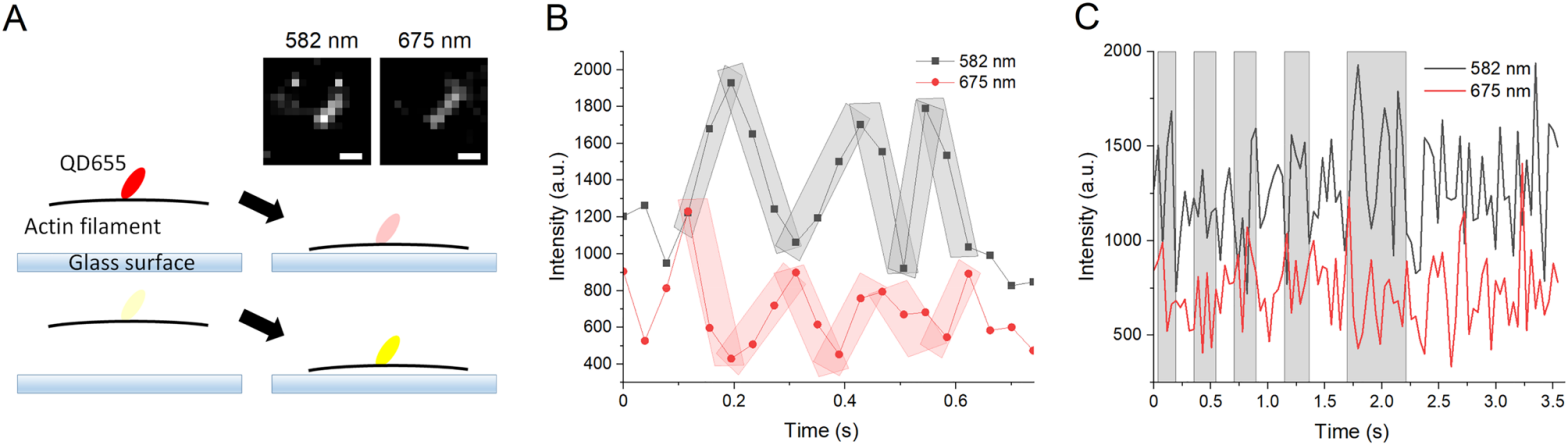
Emission intensity changes of a single Qdot655 attached on an actin filament. (**A**)(**a**) Fluorescence images (10 μm × 10 μm) of actin filaments and Qdots through a filter at 582 nm and 675 nm. The arrow shows the Qdot on an actin filament before rhodamine quenching. The emission intensities of the QD were measured after the quenching of the rhodamine fluorescence of the filament, and are shown in panels B and C. A schematic illustration of the actin filament and emission spectrum of a Qdot away from the substrate (left) and near the surface (right) observed through the red (**b**) and yellow (**c**) band path filters. (**B**) Example of the trace that showed yellow (back squares) and red (red squares) emission intensities of the emission changed in an anti-phase manner. (**C**) Typical time-dependent changes in the Qdot655 emission. Shadowed areas showing the time traces show the anti-phase changes in the intensities of the yellow and red emission intensities. The right most-shadowed area is magnified in panel (**B**).

Actin filaments were loosely tethered to the surface of glass, and sparsely labeled with Qdots, as shown in Fig. 6A. In about 30% of the time lapse data the yellow and red emission fluctuated in an anti-phase manner, suggesting that the actin subunit labeled with a Qdot approached the plasma-exposed glass surface at less than 20 nm. In this region (0 nm to 20 nm) the red and yellow emission intensity will change in an anti-phase manner, as shown in Fig. 5A. In the remaining part, the yellow and red emissions fluctuated often in an in-phase manner (Fig. 6C), suggesting that the Qdots located between 20 and 100 nm were illuminated by evanescent light (the light gradually declined from the surface with a space constant of ca. 100 nm). Thus, the changes in the intensity of the yellow and red emission fluctuations can provide the high spatio-temporal resolution of the location of an actin filament above the glass surface.

## Discussion

In this study, the intensity of the red emission spectrum (near 655 nm) of Qdots decreased when Qdots approach the plasma-treated glass, and it increased when the surface was silanized. In contrast, the yellow emission intensity of the spectrum did not appear to be affected by these treatments. The single-particle analysis of Qdots on modified glass surfaces was basically consistent with the spectral analysis of the bulk behavior of Qdots. The direct manipulation of Qdots showed the intensity of the red emission intensity declined in a glass surface distance-dependent (0−20 nm) manner, and imaging of single Qdots bound to a single actin filament promises the high spatio-temporal imaging of biomolecules labeled with Qdots near the surface of glass.

Direct manipulation and displacement of Qdots from the plasma-treated glass surface suggest that the surface effect on to the red emission intensity declines in a distance-dependent manner with the space constant (< 20 nm), which is far less than the penetration depth of the evanescent field (100 nm) or wavelength of visible light (400−700 nm). The intensity of the yellow emission intensity slightly increased as Qdots approached the surface, presumably due to the increase in the intensity of the evanescent field of illumination^24^ (Fig. 5A, upper panel).

Imaging of Qdots bound to a single actin filament showed that yellow and red emission intensities fluctuated in an anti-phase manner, suggesting the Qdot-labeled actin protomer fluctuated near the glass surface (less than 20 nm from the surface). Analysis of the Qdot emission will enable the high spatio-temporal imaging of the conformation changes in the Qdot-labeled biomolecule. This study provides an important basis for the high-resolution imaging of functioning biological molecules.

The direction dependent changes in the amplitude of the emission from a fluorophore, e.g., Qdots are reported in the preceding study by Goldman YE and colleagues^26^; they also proposed a method to estimate the direction of the fluorophore in 3D space. The direction of the longitudinal axis of individual Qdots^27^ is different and is illuminated by the polarized laser light along y-axis, which excites the Qdot most effectively when the Qdot longitudinal axis is aligned to the y-axis and less effectively when it aligned to the x-axis. This would make the photoluminescence intensity of individual Qdot different. The yellow and red emission intensities were found to vary similarly with the displacement irrespective of the photoluminescence intensity (i.e., the direction of individual Qdot longitudinal axis) as shown in Figs. 3 and 4. In the future, if accurate estimation of the direction of the Qdot's longitudinal axis and simultaneous measurement of the yellow and red emission intensities of the same Qdot are carried out, it will be possible to quantitatively estimate the distance between the Qdot and the glass surface.

The surface of glass treated by low-temperature plasma contains C, O, and Si elements, and the surface morphology of glass has not changed^21^. The strengthening effect of low-temperature plasma on the water contact angle is suggested to be mainly caused by hydroxyl and carboxyl groups^21^. Plasma treatment decreased the contact angle in this study, indicating an increase in the density of silanol groups^28^. Hydroxylation of the surface provides electrons to form hydrogen bonds with water molecules, reducing the contact angle of water droplets^21^, and may affect the emission of Qdots (see below).

At present, the exact mechanism of the glass surface distance-dependent spectral change in Qdot emission is not known. However, energy transfer from Qdots to the glass surface may explain our experimental observations, as proposed with Qdots and gold nanoparticles^14^. The distance-dependent quenching in this study is compatible with a Förster-type process. The silane layer thickness is estimated to be 27.6 nm^23^, which is near the range of the energy transfer. This value is consistent with our observation that 27 nm displacement of Qdots changed the red emission intensity, and we found this quenching was reversible and observed repetitively (Fig. 5C). Similar spectral changes were detected in all the Qdots used in this study, Qdot565, Qdot585, Qdot605, Qdot655, and Qdot705, suggesting the surface distant-dependent effects to the Qdot might be a general phenomenon in CdSe (Qdot565, Qdot585, Qdot605, and Qdot655) and CdTe (Qdot705) core Qdots.

Photoluminescence and photoluminescence excitation spectroscopy measurements of CdSe/ZnS nanocrystals (QD560)^2^ shows photoluminescence intensity between 250 and 650 nm; for the excitation (280 nm) three emission peaks are observed at 280, 400 and 560 nm; i.e., the photoluminescence intensity increases around 400 nm and 280 nm, and these peaks are less clear and are presumably originated from the emission from ZnS shell and core–shell system^2^. Photoluminescence spectra of QD655 (i.e., CdSe/ZnS nanocrystal) for different excitation wavelengths, showing a main peak at 655 nm and less clear peaks 450 nm; similar photoluminescence spectra of QD565 was also obtained (supplemental Fig. S2). The decrease in the amplitude of the 655 nm peak (band-edge emission) does not affect the amplitude of the emission between 545 and 600 nm (Fig. 1A), suggesting that the origin of the emission between 545 and 600 nm is not dependent on the band-edge emission. The origin of the emission between 545 and 600 nm is not exactly known at present, but it is worth to propose a possibility that Qdots (QD560, QD565 and QD655) shear the similar emission mechanism from ZnS shell and core–shell system^2^, which suggests that the photoluminescence intensity increase at shorter wavelength (around 550 nm) in Fig. 1A might be due to the complex emission from ZnS shell and core–shell system.

It may also be worth pointing out that the electron provided by hydroxylation may affect the emission spectra of Qdots, as proposed in the preceding study^3^; surface hydroxylation provides electrons and it quenches Qdots. This idea is consistent with the following observations. The low-pressure plasma treatment decreased the intensity of 655 nm emission of Qdots on the glass. The silanization of the glass surface increased both the contact angle of water droplets and the intensity at 655 nm emission. The emission intensity of single Qdots at 582 nm increased slightly when the distance was shortened, probably due to the increase in the evanescent field intensity^24^, and the electron transfer from the hydroxylation-provided electron may not affect the emission. A recent study^16^ found that the spectrum consists of multiple components and certain components are selectively affected by electron transfer and others are not. The electron transfer from the glass surface may affect Qdots in the same way in this study; 655 nm emission is affected by the electron transfer, but 582 nm one is not. Different mechanisms of spectrum changes in Qdots are also proposed^5,16,29^. It might be worth adding that the ion density distribution estimated by molecular dynamics simulations decays exponentially with distance from the surface (a few nm)^30^, suggesting that ion density may not be responsible for the observed decay of the red emission.

## Materials and methods

### Plasma treatment of the surface of the cover glass

Cover glass (No.1S, 40 × 50 mm: Matsunami Glass) cut into 5 mm squares was subjected to plasma treatment (0, 1, 3, 10, 30, 90 s) using a tabletop low-pressure plasma treatment system (YHS-R: Sakigake Semiconductor, Japan) to remove dust and dirt from the cover glass surface and to hydrophilize the surface.

The glass surface was silanized using dimethyldichlorosilane; the glass surface was treated with 0.01 to 10% 3-(triethoxysilyl) propyl isocyanate (Wako, Japan) and 10 mM acetic acid in 50% ethanol at 60 °C for 5 min.

Five different commercially-available core–shell Qdots were used; Qdot565, Qdot585, Qdot605, Qdot655, and Qdot705 with a ZnS shell, and a CdSe (Qdot565, Qdot585, Qdot605, and Qdot655) and CdTe (Qdot705) core, respectively (Invitrogen, USA). QD655 and QD705 were labeled with streptavidin. Qdot655 (ca. 10 nm longitudinal axis and ca. 5 nm diameter)^27^ was diluted 10 times and 2.5 μl of Qdot655 solution was dropped on the glass surface. The spectra of the Qdots were examined after evaporation of the water solvent (10 min) using an FP-8200 fluorescence spectrophotometer (equipped with a one-drop measurement unit, JASCO Corporation, Japan), with excitation from 460 to 530 nm (Fig. 1) and emission bandwidths of 5 nm at 25 °C. When an emission spectra of a plasma-treated glass piece alone without Qdots was measured with the same fluorophotometer, no emission was detected, although 5% of excitation light was reflected at the surface of the glass and slightly scattered at the surface. These imply that the photoluminescence intensity increases at shorter wavelength (around 550 nm) was successfully detected with the fluorescence spectrophotometer.

### Measurement of the contact angle

The contact angle of the glass surface and the edge of a water droplet before and after low-pressure plasma treatment was measured using a horizontally-placed binocular (Leica, MZ9s) and CCD camera (Wraymer, FLOYD-2A). The measuring principle is shown in Fig. 2. The contact angle, theta (θ), is calculated by measuring the droplet diameter (2 × r) and the spherical crown height (h) according to the following equation as mentioned by^21^;

$${\uptheta } = 2 \cdot \tan^{ - 1} \theta 1$$ and $$\tan \theta 1 = {\text{h}}/{\text{r}}.$$

### Single Qdots imaging

A Nikon inverted microscope (Ti_2_) equipped with a high numerical aperture objective (Nikon Plan 100 × NA 1.49), quad band exciter (Semrock, FF01-390/482/532/640–25), dichroic mirror (Semrock, Di03-R405/488/532/635-t1-25 × 36), bandpass filter (Semrock, FF01-446/510/581/703-25) and the perfect focus system (PSF-2) was used to image single Qdots on the modified glass surface. A solid diode laser (532 nm, CNI laser Changchun, China) was used to illuminate Qdot655. The Qdot was diluted 10^3^ times with DW and β-mercaptoethanol (10 mM), dropped on the glass surface, and observed with a total internal reflection fluorescence microscope passed through W-view (Hamamatsu photonics, Japan) equipped with two-band pass filters (Semrock, FF01-582/64 and Semrock, FF02-675/67), and projected on to an iXon Ultra 897 CCD camera (Andor, UK). For the fast image acquisition, 4 × 4 binning was applied to the full-size image, and the actual size of each pixel was 640 by 640 nm. The single Qdot used in this study showed single-step quenching as shown in the supplemental figure S3, which confirms the detection of single Qdots. The longitudinal axis of each Qdot^27^ is different and is illuminated by the polarized laser light along y-axis, which excites the Qdot most effectively when the Qdot longitudinal axis is aligned to the y-axis, and less effectively when it aligned to the x-axis^26^. For this reason, the intensity of individual Qdots in the Figs. 3 and 4 is different. A linear flexure actuator (P-603-sS1, PI Germany) or a cylindrical piezo actuator (diameter 5 mm length 25 mm, Fuji Ceramics Corporation, Japan) was used to displace the glass tip (diameter 1 µm) with Qdots as illustrated in Fig. 5 for the experimental setup. The linear flexure actuator and the cylindrical piezo actuator were used to repetitively move Qdots 27.7 nm and 60 nm up and down every one sec respectively using one Hz triangular wave signals. Light scattering from Qdots was completely blocked by the filter set (Semrock, the above dichroic mirror, the bandpass filter) and the set in W-view (Semrock, FF640-FDi0 1–25 × 36, FF01-582/64 and FF02-675/67). Single Qdot imaging was employed to exclude the possibility of contamination of the different size Qdots with different optical properties, which often makes the interpretation of experimental data of ensemble Qdots complex and difficult.

### Imaging a single Qdot on a single actin filament

Rhodamine-labeled rabbit skeletal muscle-actin (93% non-labeled: 6% Rhodamine-labeled actin: 1% biotin-labeled, Cytoskeleton Inc. US) at 0.42 mg/ml was polymerized in F-buffer (100 mM KCl, 2 mM MgCl_2_, 0.2 mM ATP, 0.1 mM DTT, 10 mM Hepes (pH 7.5) and β-mercaptoethanol (10 mM) for 12 h at 4 °C, diluted 10^4^ to 10^6^ times just before the experiment.

The glass surface treated with plasma (30 s) was conjugated with anti-biotin antibody (100 μg/ml), blocked by PLL-PEG(2) (1 mg/mL, SuSoS, Swiss), and washed with F-buffer before use. The actin filaments were put on the glass surface and those actin filaments attached to the substrate after washing with F-buffer were used for analysis. The filaments were tethered to the substrate intermittently at about 1 µm^18^.

A streptavidin conjugated Qdot655 was attached to the actin filaments (1–5 µm) and they were tethered to the anti-biotin antibody conjugated glass surface (Fig. 6). Images were recorded every 100 ms. Rhodamine-labeled actin (Cytoskeleton Inc. US) was used because rhodamine was quenched faster than Qdots under our condition, which gave more chances to obtain images from a Qdot on a single actin filament. Statistical analysis was performed with the software Origin Pro2020. The time lapse imaging was made after the quenching of rhodamine fluorescence.

## Supplementary Information

Supplementary Figures.

## Data Availability

The data that supports the finding of this study are available from the corresponding author upon request.
