# Supplementary material for: Surface-dependent quenching of Qdot emission can be a new tool for high resolution measurements
Source: Sci Rep. 2023 Feb 1;13:1869. doi: 10.1038/s41598-023-28910-8 (PMC9892493; doi:10.1038/s41598-023-28910-8)
Supplement: Supplementary file 1 — Supplementary Figures. [file 41598_2023_28910_MOESM1_ESM.pdf]

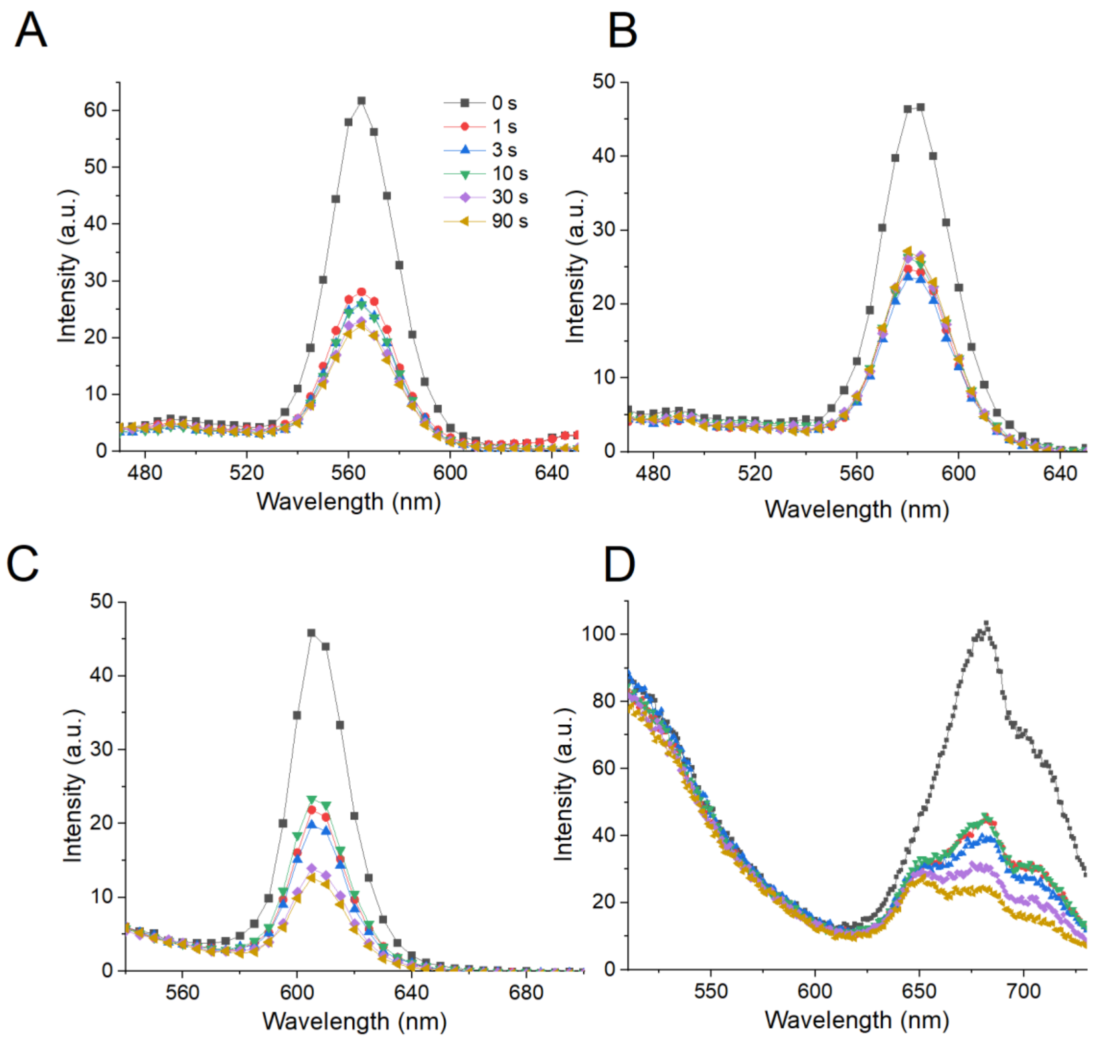

Figure S1. The effect of glass surface plasma treatment on the emission spectrum of QD565 (A), QD585 (B), QD605 (C), and QD 705 (D). Excitation at 460 nm (A and B), 530 nm (C) and 470 nm (D). Notation of the figure is the same as Fig. 1a.

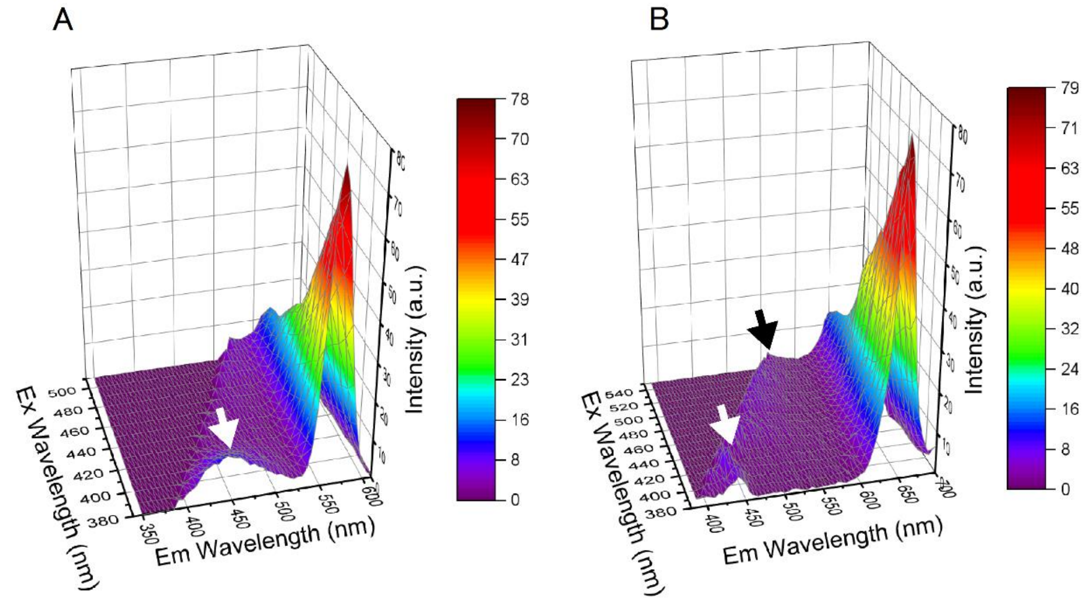

Figure S2. Three-D photoluminescence spectra of QD655 and QD565 obtained for different excitation wavelengths. Panel A shows photoluminescence spectra of QD565. The main peak at 565 nm and less clear lower peak at 450 nm is shown by the white arrow. Panel B shows that of QD655 with main peak at 655 and less clear lower peak at 450 nm is shown by the white arrow. The photoluminescence increase around 540-600 nm is shown by the black arrow.

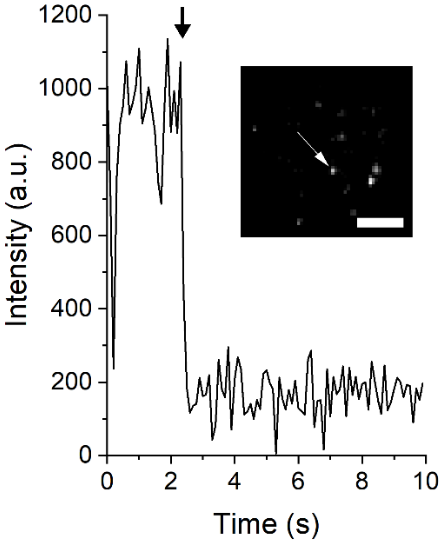

Figure S3. Single Qdot photoluminescence quenched in a single step. The fluorescence intensity of the Qdot fluorescent spot shown by an arrow in the inset quenched at the time indicated by the black arrow to the basal level. Inset shows the TIRF image of single Qdot attached on the glass surface. Bar 5 micro meter.
